# Risk Factors for Extended Duration and Timing of Peak Severity of Hot Flashes

**DOI:** 10.1371/journal.pone.0155079

**Published:** 2016-05-05

**Authors:** Rebecca Lee Smith, Lisa Gallicchio, Susan R. Miller, Howard A. Zacur, Jodi A. Flaws

**Affiliations:** 1 Department of Pathobiology, University of Illinois College of Veterinary Medicine, Urbana, Illinois, United States of America; 2 The Prevention and Research Center, The Weinberg Center for Women’s Health and Medicine, Mercy Medical Center, Baltimore, Maryland, United States of America; 3 Department of Gynecology and Obstetrics, Johns Hopkins University School of Medicine, Baltimore, Maryland, United States of America; 4 Department of Comparative Biomedicine, University of Illinois College of Veterinary Medicine, Urbana, Illinois, United States of America; VU University Medical Center, NETHERLANDS

## Abstract

**Objective:**

To identify risk factors associated with the duration of hot flashes and the time of peak hot flash severity in mid-life women.

**Methods:**

A cohort of 647 women reporting hot flashes were followed for 1–7 years, with survey data and hormone measurements. Survival analysis determined the association of risk factors with the duration of hot flashes. Linear regression determined the association of risk factors with the time of peak severity. Final models were determined through stepwise model selection.

**Results:**

Average hot flash duration was 2.5 years (range: 1–33), with peak severity on average at 2.96 years (range: 1–20). Duration of hot flashes was associated with race, education, menopause status, smoking history, BMI, alcohol consumption, leisure activity levels, and levels of estradiol and progesterone. In the final model, only race, alcohol consumption, leisure activity, and menopause were retained. White women had significantly shorter hot flash durations than non-white women. Women consuming at least 12 alcoholic drinks in the previous year had a significantly shorter duration of hot flashes with a smaller effect of hot flash duration on increasing in time to peak severity compared to those who consumed less than 12 alcoholic drinks in that year. Higher serum progesterone levels were associated with later peak severity if the duration of the hot flashes was less than 2 years and an earlier peak severity otherwise.

**Conclusions:**

These results suggest that some behaviors (such as moderate alcohol consumption) are associated with shorter durations of hot flashes, and that progesterone was associated with the dynamics of hot flash severity.

## Introduction

Approximately 75% of perimenopausal women experience hot flashes;[1] this percentage has been shown to vary by certain demographic (i.e. ethnicity[2]) and behavioral factors (i.e. smoking,[3] exercise,[4] or body mass index[5]). Hot flashes are associated with increases in medical costs due to treatment (prescription, over-the-counter, alternative, and dietary), physician visits, laboratory testing, and loss of productivity.[6] There is also a decrease in perceived quality of life[7] during the perimenopausal period in women with such symptoms. It is not well understood, however, how the symptoms change during the menopausal transition.

The existing knowledge on the risk factors of hot flash dynamics is limited. Most previous studies of hot flashes have been cross-sectional[8–10], and thus, provide little information on the temporality of the relationship and no opportunity to examine changes over time. Others assess the outcome of hot flashes using only one question, providing an incomplete picture of the hot flash experience. For instance, other cohort studies have asked about the presence of symptoms over limited time periods[11–13], which does not capture information on severity or allow for examination of changes across the year between surveys. Further, most studies looking at changes over time have focused on treatment efficacy[14], not the dynamics of untreated hot flashes. A recent study on dynamics without treatment reported interesting associations between hot flash duration and a number of potential risk factors, but this study was focused on frequent hot flashes, rather than all hot flashes, and did not investigate the dynamics of hot flash severity.[11] Another study looked primarily at hormonal changes, not at the underlying risk factors or at the duration of symptoms.[15] Filling the gap in knowledge regarding changes over time in the hot flash experience is critical, as two of the most common questions that perimenopausal women will ask is: how long will hot flashes last and when will they start to improve?

The purpose of this study was to examine the duration of hot flash symptoms in US women in mid-life, and to identify risk factors for longer durations. A secondary purpose was to examine the time to peak severity of hot flash symptoms and identify factors affecting that time. By studying the relationship between risk factors and changes in symptoms, we will be able to identify actions that can be taken before and during the menopause transition to improve clinical outcome.

## Methods

All participants gave written informed consent according to procedures approved by the University of Illinois and Johns Hopkins University Institutional Review Boards. University of Illinois and Johns Hopkins University Institutional Review Boards approved this research. The study design for the parent study is described in detail elsewhere.[5] Briefly, a cohort study of hot flashes among women 45–54 years of age was conducted starting in 2006 among residents of Baltimore and its surrounding counties. Women were recruited by mail, and were included if they were in the target age range, had intact ovaries and uteri, and were pre- or perimenopausal. Exclusion criteria consisted of pregnancy, a history of cancer, exogenous female hormone or herbal/plant substance, and no menstrual periods within the past year. Participants made a baseline clinic visit, which included measurement of height and weight to calculate body mass index (BMI) and completion of a detailed 26-page baseline survey. Participants were asked to complete a brief questionnaire during a clinic visit 3 weeks after the baseline visit, then annually after that the baseline visit. This questionnaire repeated all previous questions about hot flashes and smoking, and BMI was calculated during the visit. Blood samples were collected at each scheduled clinic visit and stored until measurement of hormone levels as described below. Menopausal status was defined as follows: premenopausal women were those who experienced their last menstrual period within the past 3 months and reported 11 or more periods within the past year; perimenopausal women were those who experienced 1) their last menstrual period within the past year, but not within the past 3 months, or 2) their last menstrual period within the past 3 months and experienced 10 or fewer periods within the past year; postmenopausal women were those women who had not experienced a menstrual period within the past year. Follow-up was discontinued for women if they reported hormone therapy, an oophorectomy, or a cancer diagnosis. At the year 4 visit, follow-up was discontinued for women determined to be postmenopausal. This analysis included all participants enrolled as of February 2015, and consisted of the information gathered in the baseline survey and in the annual follow-up surveys.

During the baseline survey, women were asked if they had ever had hot flashes, with a description of hot flashes provided. They were also asked how old they were when they first experienced hot flashes, how old they were when the hot flashes were most severe, and the severity of the majority of their hot flashes. During annual follow-up visits, women were asked if they had hot flashes since their last visit, and were again asked the severity of the majority of their hot flashes. In terms of severity, each woman was asked at each visit to describe her hot flashes as: mild (sensation of heat without sweating), moderate (sensation of heat with sweating), or severe (sensation of heat with sweating that disrupts usual activity).

Serum samples extracted from the collected blood samples were used to measure estradiol, and progesterone levels in each sample using commercially available, previously validated enzyme-linked immunosorbent assay (ELISA) kits (DRG, Springfield, New Jersey, USA).[16–19] The minimum detection limits and intra-assay coefficients of variation were as follows: estradiol 7 pg/ml, 3.3 ± 0.17%; testosterone 0.04 ng/ml, 2.2 ± 0.56%; and progesterone 0.1 ng/ml, 2.1 ± 0.65. The average inter-assay coefficient of variation for all assays was less than 5%. In the case of values lower than the detection limits for the assay, we used the limit of detection as the hormone value. Each sample was measured in duplicate within the same assay. Progesterone, testosterone, and estradiol levels were log-transformed to meet normality assumptions.

### Duration

Only women who reported hot flashes at any point during the study were included in this analysis. Duration of hot flashes was determined by the difference in the age at first hot flashes (self-reported) and the age at which the woman first reported no longer experiencing hot flashes. Specifically, the end of hot flashes was assumed to occur in the year prior to the first year a woman reported no hot flashes. If women later reported a resumption of hot flashes, the duration of hot flashes was calculated to include the full length of time, including the years in which no hot flashes were reported. Women who reported an end to hot flashes were interval censored; women who did not report an end to hot flashes during the study were right-censored. Parametric accelerated failure time models were fit based on Weibull and exponential distributions, with the best-fitting model (based on Bayesian Information Criterion, BIC) used as a base for all other analyses.

Risk factors considered were education level, race, history of pregnancy, menopause status, smoking history, BMI, alcohol use (at least 12 drinks in an average year, at least 12 drinks in the last year, the number of days in which alcohol was consumed in the last year, or the total estimated number of drinks consumed in the previous year), relative activity level during leisure (a 5-point Likert scale from ‘much less active’ to ‘much more active’), relative activity level during work (a 5-point Likert scale from ‘much lighter’ to ‘much heavier’), estradiol levels, testosterone levels, and progesterone levels. A combined variable for total exercise, being the sum of the leisure and work activity levels, was also considered; for women not reporting work activity levels, the leisure activity level was doubled as it was assumed that the women spent twice the amount of time at leisure. Race, education, and history of pregnancy were considered constant variables, and were determined by the first survey. All other variables were considered time-dependent, and were allowed to vary between years as determined by follow-up survey responses. Continuous variables were tested for normality and transformed if normality assumptions failed to hold. The counting method, with a frailty term for each woman to account for non-independence of responses within subjects, was used to account for time-dependency.

Univariate models were fit for all risk factors, and those with p≤0.1 were included for consideration in the multivariate model. BIC-based stepwise model selection was applied, in which for each step the single change (addition or subtraction) that produced the model with the lowest BIC value (a conservative variation of the ratio, with penalties for adding parameters) was accepted. All main effects and interactions considered. All models were fit with the survival package in R,[20] accessed through Revolution R Enterprise,[21] and model selection used the stepAIC function in the MASS package.[22]

### Peak Severity

Time to peak severity was taken as the difference between the age at which hot flashes were most severe and the age at which hot flashes were first experienced. Peak severity was assumed to be the age at which hot flashes were reported to be most severe during the baseline visit, unless a woman later reported a higher severity than reported at the baseline visit; in that case, the age at which the woman first reported the higher severity was considered to be the peak. Peak severity varied in intensity, with some women having only mild or moderate hot flashes during their peak. All risk factors considered (education level, race, history of pregnancy, menopause status, smoking history, BMI, alcohol use, relative activity level during leisure, relative activity level during work, and estradiol, testosterone, and progesterone levels) consisted of their baseline values, with no time-dependent variables. The duration of the hot flashes, as calculated above, was also included as a risk factor. Linear regression models for the time to peak severity, in years, were fit with the glm function in R, with model selection procedures as described above.

## Results

**Fig 1:**
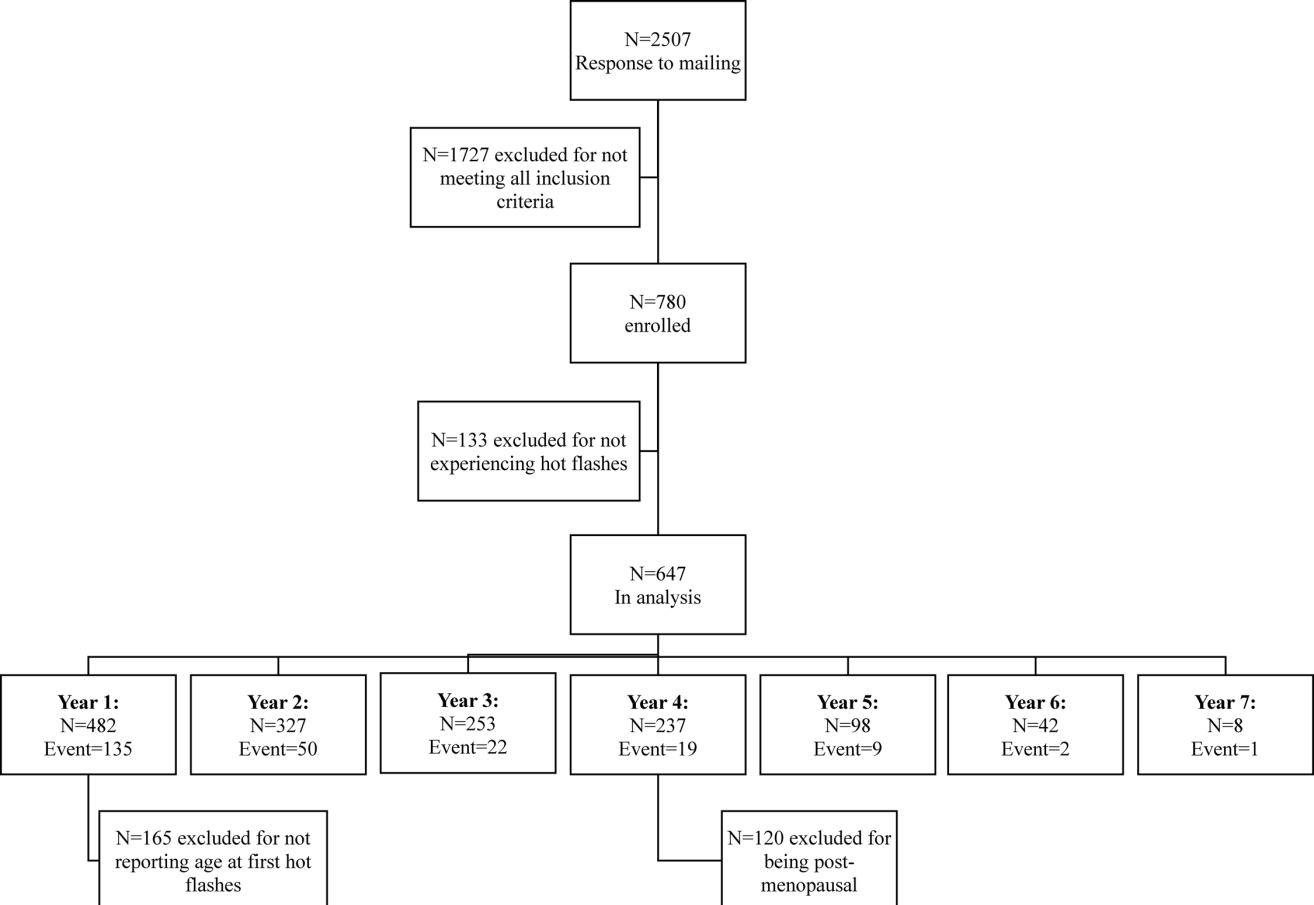
Flow chart of women enrolled in the study. Event indicates that hot flashes ended during that year.

A total of 238 women reporting hot flashes also reported the end of those hot flashes during the study, with 482 reporting the time of their first hot flashes, out of 647 women reporting hot flashes in the total cohort of 780 women. The number of women enrolled in the study and participating in each year is demonstrated (Fig 1); at time of enrollment, 75 women had not yet experienced hot flashes, 320 women had hot flashes for 1–5 years, 55 women had hot flashes for 5–10 years, 17 had hot flashes for 10–15 years, and 15 had hot flashes for more than 15 years. Women were followed for a median of 4 years (range: 2 to 7). Among women reporting an end to hot flashes, the range of duration of hot flashes was reported as 1 to 29 years, with a mean of 2.5 years and a median of 1 year; among all women experiencing hot flashes (including those for whom those hot flashes had not ended at the time of the study), the mean duration of hot flashes at the time of the study was 6 years with a median of 4 years. Of the women experiencing hot flashes more than 20 years, 4 began experiencing hot flashes as teenagers, 2 began in their 20s, 4 began in their 30s, 4 began in their 40s, and one began at the age of 53. The range of time to peak severity of hot flashes was reported as 0 to 20 years, with a mean of 2.97 years and a median of 2 years; as this included women whose hot flashes had not ended, the median is greater than the median duration of women reporting an end to hot flashes. These numbers are presented by category of each covariate in Table 1. Two women who reported an age at peak severity prior to their age at first hot flashes were removed from the analysis.

**Table 1:** Description of data used to study the effects of various risk factors on the duration of hot flashes in US women aged 45–54.

Parameter	Level	Women with hot flashes	Women with observed end to hot flashes	Mean duration of hot flashes (range) in years	Mean time to peak severity (range) in years	p-value^*^ for duration	p-value^†^ for time to peak severity
**Race**	**White**	421	164	2.32 (1–25)	2.75 (0–20)	0.06	0.08
	**Non-White**	224	73	2.93 (1–29)	3.5 (0–14)		
**Education**	**Graduated College**	404	156	2.32 (1–29)	2.96 (0–20)	0.03	0.38
	**Did Not Graduate College**	241	81	2.83 (1–26)	2.98 (0–20)		
**Ever Pregnant**	**Yes**	574	208	2.54 (1–29)	2.93 (0–20)	0.21	0.52
	**No**	72	30	2.23 (1–18)	3.25 (0–16)		
**Menopause Status**^**‡**^	**Pre**	339	133	2.23 (1–25)	2.53 (0–15)	<0.001	0.16
	**Peri**	483	80	2.58 (1–29)	3.07 (0–20)		
	**Post**	236	24	3.88 (1–12)	4.12 (0–18)		
**Smoking**^**‡**^	**Current**	151	18	3.89 (1–29)	3.05 (0–20)	0.002	0.92
	**Former**	369	86	2.52 (1–26)	2.95 (0–20)		
	**Never**	467	134	2.31 (1–25)	2.97 (0–19)		
**BMI**^**‡**^	**<25**	376	95	2.48 (1–29)	2.42 (0–19)	<0.001	0.02
	**25–30**	314	72	2.36 (1–26)	3.33 (0–20)		
	**>30**	366	71	2.68 (1–25)	3.35 (0–19)		
**Alcohol Consumed**^**‡**^	**≥12 Drinks/ Year**	536	171	2.44 (1–29)	2.47 (0–20)	<0.001	<0.001
	**<12 Drinks/ Year**	291	48	3.12 (1–25)	4.78 (0–20)		
**Leisure Activity**^**‡**^	**Much Less**	51	9	1.22 (1–3)	4.33 (0–14)	0.03	0.82
	**Less**	190	39	1.92 (1–14)	2.82 (0–20)		
	**As Much**	251	59	1.78 (1–12)	3.13 (0–19)		
	**More**	176	36	1.86 (1–26)	3.61 (0–11)		
	**Much More**	66	19	1.16 (1–3)	1.81 (0–19)		
**Work Activity**^**‡**^	**Much Less**	116	25	1.92 (1–13)	3.54 (0–11)	0.76	0.21
	**Less**	244	56	1.96 (1–14)	3.12 (0–14)		
	**As Much**	235	53	1.57 (1–26)	3.1 (0–20)		
	**More**	91	16	2.12 (1–7)	3.22 (0–19)		
	**Much More**	24	8	1.62 (1–6)	2.18 (0–12)		
**Estradiol Levels**^**‡**^^**^**^^**#**^	**<42**	493	110	2.2 (1–13)	3.11 (0–20)	<0.001	0.10
	**≥42**	427	128	2.77 (1–29)	2.79 (0–20)		
**Progesterone Levels**^‡^^**^^#^	**<0.2**	497	92	2.35 (1–13)	3.28 (0–19)	<0.001	0.09
	**≥0.2**	442	146	2.6 (1–29)	2.67 (0–20)		
**Testosterone Levels**^‡^^**^	**<0.34**	442	95	3.04 (1–29)	3.07 (0–20)	0.18	0.96
	**≥0.34**	483	143	2.15 (1–26)	2.9 (0–19)		
**Total**		**647**	**238**	**2.50 (1–29)**	**2.97 (0–20)**		

^*^p-values for duration are based on an accelerated failure time model with exponential distribution and interval censoring.

^†^p-values for time to peak severity are based on a linear regression model with baseline data for all covariates.

^**‡**^These parameters are time-dependent, and may vary throughout the study. Therefore, the sum of the number of women in each level of a parameter may be greater than the total number of women in the study.

^**^These parameters are included as continuous variables in the analysis; levels for this table were chosen based on the median value in the sample.

^#^These parameters were log-transformed for statistical analysis.

### Duration

Progesterone and estradiol levels were log-transformed to meet normality assumptions. Race, education level, menopause status, smoking history, BMI, alcohol use, relative activity during leisure, exercise level, progesterone levels, and estradiol levels were all significantly related with the duration of hot flashes (Table 1). For alcohol use, the number of days on which alcohol was consumed in the last year and the estimated amount of alcohol consumed in the last year were not significantly associated with the duration of hot flashes, but both other variables (whether at least 12 drinks were consumed in the last year or in an average year) were associated with hot flashes. As the variable representing consuming at least 12 drinks in the last year was more strongly associated with the outcome, it was included in the model selection process.

**Fig 2:**
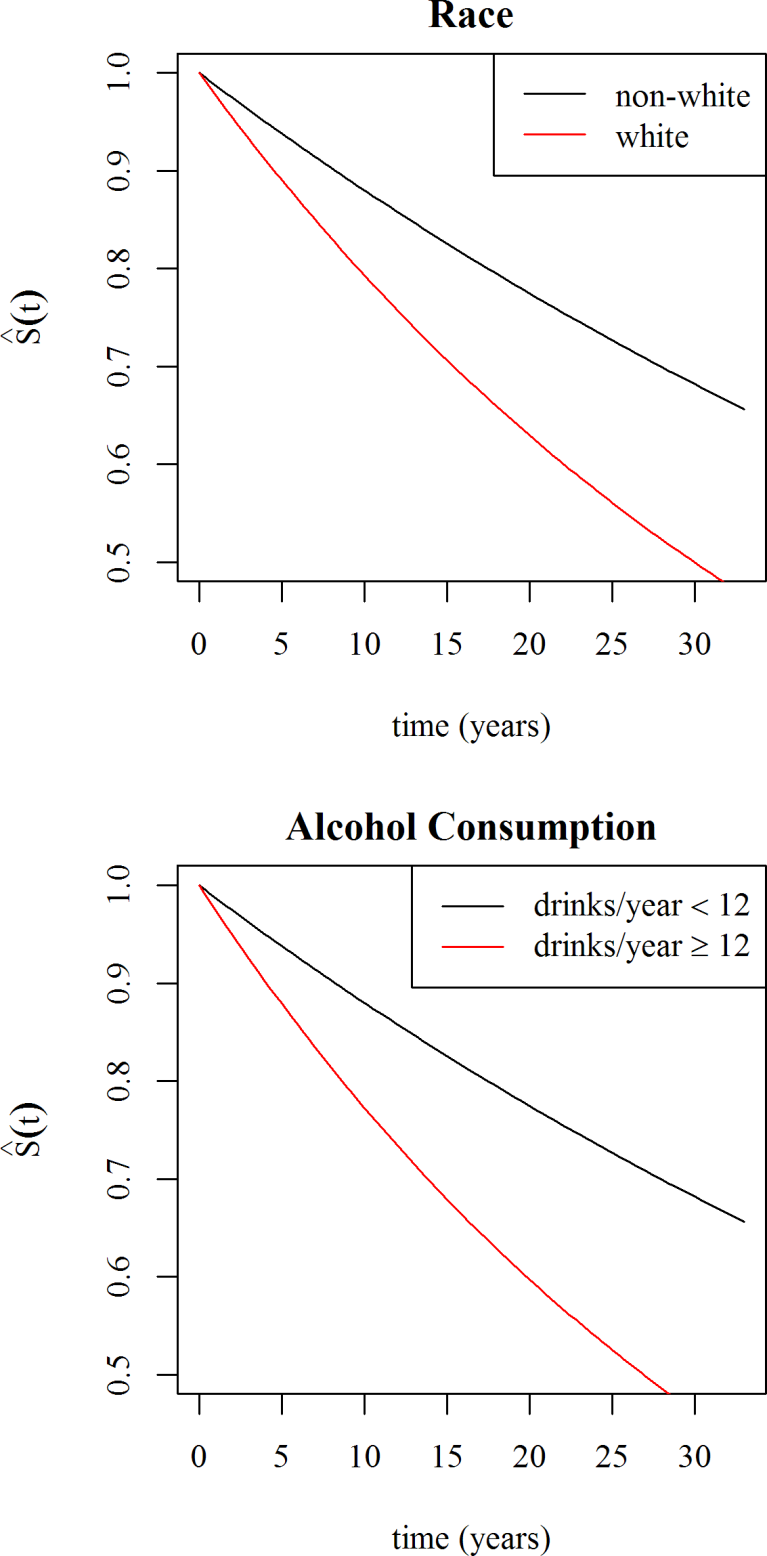
Survival curves for duration of hot flashes in perimenopausal US women by race (top) and alcohol consumption (bottom). Survival at time t, S(t), is defined as still experiencing hot flashes t years after their onset. Race was divided into white (red line) and non-white (black line). Alcohol consumption was divided into <12 drinks in the last year (black line) and at least 12 drinks in the last year (red line).

In the final multivariate model, no interactions were included; smoking, BMI, exercise level, and progesterone and estradiol levels were removed during the stepwise selection process. The results of the final model are shown in Table 2, and projected survival curves are shown (Fig 2). White women experienced hot flashes for significantly shorter durations than non-white women. Women who had consumed at least 12 drinks in the previous year had shorter durations of hot flashes than those who had not consumed at least 12 drinks in the previous year. Although leisure activity was retained in the model, the effect was not significant either statistically or practically.

**Table 2:** Final model for duration of hot flashes in US women^*^.

Parameter	Level	Coefficient	Standard Error	p-value
**Intercept**		4.36	0.27	<0.001
**Race (non-white is base)**	**white**	-0.59	0.20	0.002
**Alcohol use (<12 drinks in last year is base)**	**≥12 drinks in last year**	-0.70	0.22	0.002
**Menopause status (perimenopausal is base)**	**Postmenopausal**	0.73	0.41	0.07
	**Premenopausal**	-1.43	0.18	<0.001
**Level of Leisure Activity**		-0.03	0.08	0.70

^*^ Results are from an accelerated failure time model, with exponential distribution and interval censoring, using BIC-based stepwise model selection.

The effect of menopause status on hot flash duration (postmenopausal women experience hot flashes for longer periods of time than premenopausal women) is an artifact of the counting method; 39% of premenopausal data points are censored (35% experiencing the end of hot flashes in postmenopause and the remaining experiencing the end of hot flashes in perimenopause), compared to 17% of perimenopausal and 10% of postmenopausal (see Table 1), and postmenopausal women who are still experiencing hot flashes have been experiencing them for a longer period of time. This is an expected result of using a variable of this nature.

In the study, 15 women experienced hot flashes for more than 20 years. A subset analysis of the data excluding those women produced similar results for race, alcohol use, menopause status, and leisure activity. The final model also included progesterone and an interaction between progesterone and menopause status; increases in progesterone decreased duration in premenopausal women and increased duration in peri- and post-menopausal women.

### Peak Severity

Hot flash duration, BMI, alcohol use, race, and progesterone levels were all significantly related with the time to peak hot flash severity. In the final multivariate model (Table 3), BMI and race were removed. Interactions were added for duration with both alcohol use and progesterone levels. As the duration of hot flashes increased, the time of peak hot flash severity also increased; however, the increase was more gradual in women who consumed at least 12 alcoholic drinks in the previous year. As progesterone levels increased, the time to peak hot flash severity increased in women with hot flash duration of up to 2 years. In women experiencing hot flashes for longer than 2 years, increasing progesterone levels decreased the time to peak hot flash severity.

**Table 3:** Final model^*^ for years to peak hot flash severity in US women.

Parameter	Coefficient	Standard Error	p-value
**Intercept**	-0.39	0.44	0.376
**Hot Flash Duration (years) if Alcohol Use <12 drinks in last year**	0.72	0.05	<0.001
**Hot Flash Duration (years) if Alcohol Use ≥12 drinks in last year**	0.28	0.05	<0.001
**Alcohol use ≥12 drinks in last year**	1.41	0.49	0.004
**Log (Progesterone level) (ng/ml)**	0.16	0.12	0.171
Hot Flash Duration^*^Log (Progesterone Level)	-0.06	0.01	<0.001

^*^ Results are from a linear regression model using BIC-based stepwise model selection.

Women who consumed at least 12 alcoholic drinks in the previous year had later peaks in hot flash severity than those who did not if the duration of their hot flashes was less than 3 years; women experiencing hot flashes for longer than 3 years had earlier peak severity if they reported ≥12 drinks consumed in the previous year.

In the subset analysis excluding women who experienced hot flashes for more than 20 years, the relationship of time to peak severity with hot flash duration and alcohol use was similar. Progesterone levels were no longer included in the final model. Race was included in the final model, with white women having significantly earlier peak severity.

## Discussion

This study has found that the majority of women do not experience hot flashes for long periods of time and that certain risk factors are associated with longer hot flash duration that have not been previously identified. Race was associated with the duration of hot flashes, as it was with their likelihood, frequency, and severity:[2,23] Non-white women (in this study, the category was primarily African-American women) were more likely to have hot flashes than white women, which agrees with previous studies.[11] The reason for this association is unknown; although BMI has been suggested as a risk factor associated with race and was correlated with hot flash duration in the univariate analysis, BMI was not retained in the final model of this study. The data used in this study have shown a correlation between race and estrogen levels, however,[23] which may be another mechanism behind the association. African American women were more likely to have low estradiol levels compared to Caucasian women, and the univariate analysis showed that the duration of hot flashes decreased as estradiol levels increased, although the effect disappeared when controlling for race, alcohol consumption, menopause status, and leisure activity. It is possible that the effect of estradiol is one of the biological mechanisms through which one or all of these other factors act, and that the effect of estradiol, an intervening variable, is masked by the effect of the other factors. It should be noted that women who were given hormone treatment were excluded from this study, so the results may not include the more extreme spectrum of symptoms.

Besides the demographic risk factors, there were lifestyle choices associated with the duration of hot flashes, and consideration of these factors may help identify interventions or protective measures to be studied in perimenopausal women. We found that some amount of alcohol consumption (at least 12 drinks in the previous year, compared to less than 12 drinks in the previous year) resulted in significantly shorter hot flash duration in women. Another study found that high alcohol consumption (>16 drinks per week) was associated with increased vasomotor symptoms, but this was compared to <10 drinks per week, and so was comparing heavy consumption with lower consumption,[24] while in this study, no women reported such heavy consumption and even a more lenient definition of heavy drinking (no more than 3 drinks per day, on average) was not found to be significantly related to duration of vasomotor symptoms. Another study only examined alcohol consumption per day as a linear variable, finding that there was no effect,[2] which was supported by our univariate analysis. However, a study that looked specifically at the effects of moderate drinking compared to high or low amounts of alcohol consumption found that moderate consumption was protective against hot flashes in perimenopausal women.[25] The mechanism is unclear, possibly acting through alteration of serum estrogen or an effect on temperature regulation. It is possible that women did not consume alcohol because of other (unmeasured) health conditions which raised the risk of extended hot flashes. This suggests that there may be worth in studying the effects of moderate alcohol consumption in women suffering from hot flashes, if other health circumstances allow.

Although smoking was not retained in the final model, in the univariate analysis current and former smokers both suffered from longer periods of hot flashes than non-smokers, but former smokers were numerically (if not significantly) better off than current smokers. Therefore, quitting smoking was associated with shorter duration of hot flashes as well as less frequency and severity.[3] In addition, multiple studies have identified smoking as a risk factor for early onset of menopause.[26–30] The mechanism of this relationship is unknown, but smoking is believed to alter hormone levels by changing hormone metabolism[31–35] or to change thermoregulatory pathways.[36,37] Similar to smoking, education was not retained in the final model, but graduating from college was significantly associated with shorter hot flash duration in the univariate analysis. It is possible that this relationship was due to the correlation in these data between education and a number of other factors that were retained in the final model, such as race (p<0.001), leisure activity (p<0.001), and alcohol consumption (p = 0.07).

Leisure activity was significantly correlated with a number of other variables (education level, race, alcohol consumption, work activity, BMI, and progesterone levels), and so may have been reflecting some of the effect of a number of these. Exercise has been recommended as a method for alleviating hot flashes, but the evidence has been mixed.[38] Our results show that leisure activity must be controlled for in predicting the duration of hot flashes, but did not significantly affect the duration itself.

The model for time to peak severity found that a longer duration of hot flashes delayed that peak. However, alcohol consumption was associated with a smaller effect of the duration of hot flashes on the time to the peak severity. Both effects (shorter duration and earlier peak) may be caused by a similar process, although again the biological mechanism is unclear. Higher serum progesterone levels were associated with decreased time to peak severity in women with longer hot flash durations; low progesterone levels have been found to be associated with risk and severity of hot flashes,[39] possibly acting through thermoregulation pathways.[40] Progesterone has been used in trials as a treatment for hot flashes,[41] and these results suggest that such treatment should be studied to determine its effect on the peak (and subsequent decline) in severity. This result may be reflecting women still in the menopausal transition, as progesterone levels will be fluctuating in women who are still ovulatory; however, the timing of clinic visits without regard to menstrual cycle should have served to smooth the progesterone levels in these women, so this bias is unlikely to have occurred. In addition, forcing menopause status at baseline into the final model for time to peak severity did not change the observed relationship between progesterone levels at baseline and time to peak severity, indicating that these results were not confounded by the potential occurrence of ovulation.

One limitation to the ability of this study to understand dynamics is the periodic nature of the surveys. The duration of hot flashes is short in most women, with a highly right-skewed distribution: the median of the upper end of the duration interval in this study was 2, and the mode was 1. As such, fine differences in durations were not detectable. However, the long tail of the distribution enabled us to determine risks for the larger differences which may be more clinically relevant. The use of interval censoring improved that ability, as well, by accounting for our inability to determine the exact event time. The study is also limited by the self-reporting of severity, as a woman may change her definition of “moderate” or “severe” hot flashes as her symptoms continue. It is also possible that the study suffered from some amount of selection bias, as women undergoing treatment for their hot flashes (who might be assumed to be suffering from more severe symptoms) were excluded from the study; however, many women in the study reported severe and frequent hot flashes, so this bias is not evident. Recall bias could also affect the results of this study, as many women were suffering from hot flashes prior to the study onset and had to remember the year in which their hot flashes started; this bias is unlikely to be systematic and so should not affect the results. Despite the limitations, this data set has allowed for the most detailed analysis of hot flash dynamics to date.

One other possible limitation to these data was the high rate of censoring for the duration analysis. To determine the potential bias of the high censoring observed, sensitivity analysis was performed by repeating the analysis under 2 assumptions: that all women experienced an end to hot flashes before their last visit, or that all women not reporting an end to hot flashes experienced the maximum duration of hot flashes. Under these analyses, little change was seen in either univariate or multivariate models; therefore, we may conclude that the high rate of censoring did not bias our estimate.

This study has found that there are both demographic and behavioral risk factors associated with longer duration of hot flashes in perimenopausal women and later peaks in severity. This information may be useful to clinicians advising women on what to expect with hot flashes and potential interventions to limit their duration and severity.
